# Primary Care Detection of Chronic Kidney Disease in Adults with Type-2 Diabetes: The ADD-CKD Study (Awareness, Detection and Drug Therapy in Type 2 Diabetes and Chronic Kidney Disease)

**DOI:** 10.1371/journal.pone.0110535

**Published:** 2014-11-26

**Authors:** Lynda A. Szczech, Rebecca C. Stewart, Hsu-Lin Su, Richard J. DeLoskey, Brad C. Astor, Chester H. Fox, Peter A. McCullough, Joseph A. Vassalotti

**Affiliations:** 1 Durham Nephrology Associates, Durham, North Carolina, United States of America; 2 Scientific Activities Department, The National Kidney Foundation, Inc., New York, New York, United States of America; 3 Covance Inc., Princeton, New Jersey, United States of America; 4 Departments of Medicine and Population Health Sciences, University of Wisconsin School of Medicine and Public Health, Madison, Wisconsin, United States of America; 5 Department of Family Medicine, University at Buffalo, Buffalo, New York, United States of America; 6 Baylor Health Care System, Baylor Heart and Vascular Institute, Dallas, Texas, United States of America; 7 Department of Medicine, Division of Nephrology, Icahn School of Medicine at Mount Sinai, New York, New York, United States of America; University of Florida, United States of America

## Abstract

This US, multicenter, observational study assessed the CKD prevalence in adult patients with type-2 diabetes mellitus (T2DM) and characterized the proportion of detected and undiagnosed CKD in the primary care setting using the following: a clinician survey; a patient physical exam and medical history; a single blood draw for estimated glomerular filtration rate (eGFR) and glycosolated hemoglobin (HbA1c); urine dipstick for protein; urine albumin-creatinine ratio (ACR); two patient quality of life questionnaires; and a 15-month medical record review. The study consisted of 9339 adults with T2DM and 466 investigator sites. Of the 9339 enrolled, 9307 had complete data collection for analysis. The 15-month retrospective review showed urine protein, urine ACR, and eGFR testing were not performed in 51.4%, 52.9% and 15.2% of individuals, respectively. Of the 9307 patients, 5036 (54.1%) had Stage 1–5 CKD based on eGFR and albuminuria; however, only 607 (12.1%) of those patients were identified as having CKD by their clinicians. Clinicians were more successful in diagnosing patients with Stage 3–5 CKD than Stages 1 and 2. There were no differences in clinicians’ likelihood of identification of CKD based on practice setting, number of years in practice, or self-reported patients seen per week. Awareness or patient self-reported CKD was 81.1% with practitioner detection versus 2.6% in the absence of diagnosis. Primary care of T2DM demonstrates recommended urine CKD testing is underutilized, and CKD is significantly under-diagnosed. This is the first study to show CKD detection is associated with awareness.

## Introduction

Chronic kidney disease (CKD) is common and increasing in prevalence in the US. The estimated proportion of CKD stages 1 to 4 in US adults grew from 10% to 13.1% between 1994 and 2004, based on the National Health and Nutrition Examination Survey (NHANES) [1]. In the same ten-year interval, the US CKD population treated with dialysis or kidney transplantation increased from 270 to 468 thousand patients [2]. The increase in CKD can be accounted for primarily by the expansion of the population with the leading risk factor, T2DM, driven in turn by the obesity epidemic and the aging of the population [3]. Major adverse outcomes of CKD include progression to chronic kidney failure and impaired kidney function-related complications, such as cardiovascular disease. Individuals with impaired kidney function, albuminuria, and especially both are more likely to experience a cardiovascular event than reach end stage renal disease [4]. Since approximately two-thirds of all drugs are cleared by the kidneys, impaired kidney function has recently emerged as a major patient safety risk. The enormous economic burden of CKD is demonstrated by 18 billion dollars or 26.1% of expenditures for Medicare beneficiaries with diabetes and CKD in 2009, an 11-fold increase since 1993 [2].

The current criteria for detection of diabetes according to the American Diabetes Association (ADA) includes hemoglobin A1c (HbA1c) ≥6.5%, fasting plasma glucose ≥126 mg/dL (7.0 mmol/L), or a random plasma glucose concentration ≥200 mg/dL (11.1 mmol/L) [5]. Less well-known to primary care practitioners (PCPs) is the National Kidney Foundation Kidney Disease Outcomes Quality Initiative (NKF KDOQI) Clinical Practice Guidelines and Clinical Practice Recommendations on CKD. KDOQI defines CKD and stratifies the disease into 5 stages based on significant albuminuria or proteinuria for ≥3 months, and/or GFR <60 mL/min/1.73 m^2^ for ≥3 months [6].

Awareness of CKD remains low at 10% in US adults in part because CKD is usually silent until its late stages [7]. The diagnosis of CKD by the PCP often occurs during the later stages of CKD, when there are few opportunities to prevent adverse outcomes. Earlier detection allows more time for evaluation and treatment but requires explicit testing strategies for asymptomatic individuals at increased risk of CKD, such as those with T2DM. Significant deficiencies in the quality of CKD primary care are well documented, including a 26% use of potentially harmful drugs in one regional US study [8]. In a cross-sectional sample of US patients in 2008 that examined the onset of chronic kidney failure, only 57% received any nephrology care, while only 25% of patients were treated by a nephrologist for more than one year [9]. Observational data showing a levelling off of the incidence of chronic kidney failure from diabetes in recent years suggests that improved preventative care of T2DM can reduce the rate of progression of CKD [10]. However, the US Preventive Services Task Force does not endorse CKD screening of asymptomatic individuals because of the lack of evidence from randomized-controlled trials [11]. Therefore, studies evaluating the impact of early detection of CKD by PCPs in patients with T2DM are vital. Detection of CKD by PCPs should drive aggressive cardiovascular risk factor control and drug prescription practices that include a patient safety approach to impaired kidney function and appropriate referral to nephrology for advanced or rapidly progressing disease. This study was designed to assess the routine practices of PCPs nationwide and sensitivity of detection of CKD in real world clinical practices in adults with T2DM. The results of this study provide foundational data to address the impact of detection on outcomes. Opportunities for improvement include increasing PCP recognition, earlier treatment of CKD, and improving collaborative care with nephrology [8].

## Methods

### Study Design

**Figure 1:**
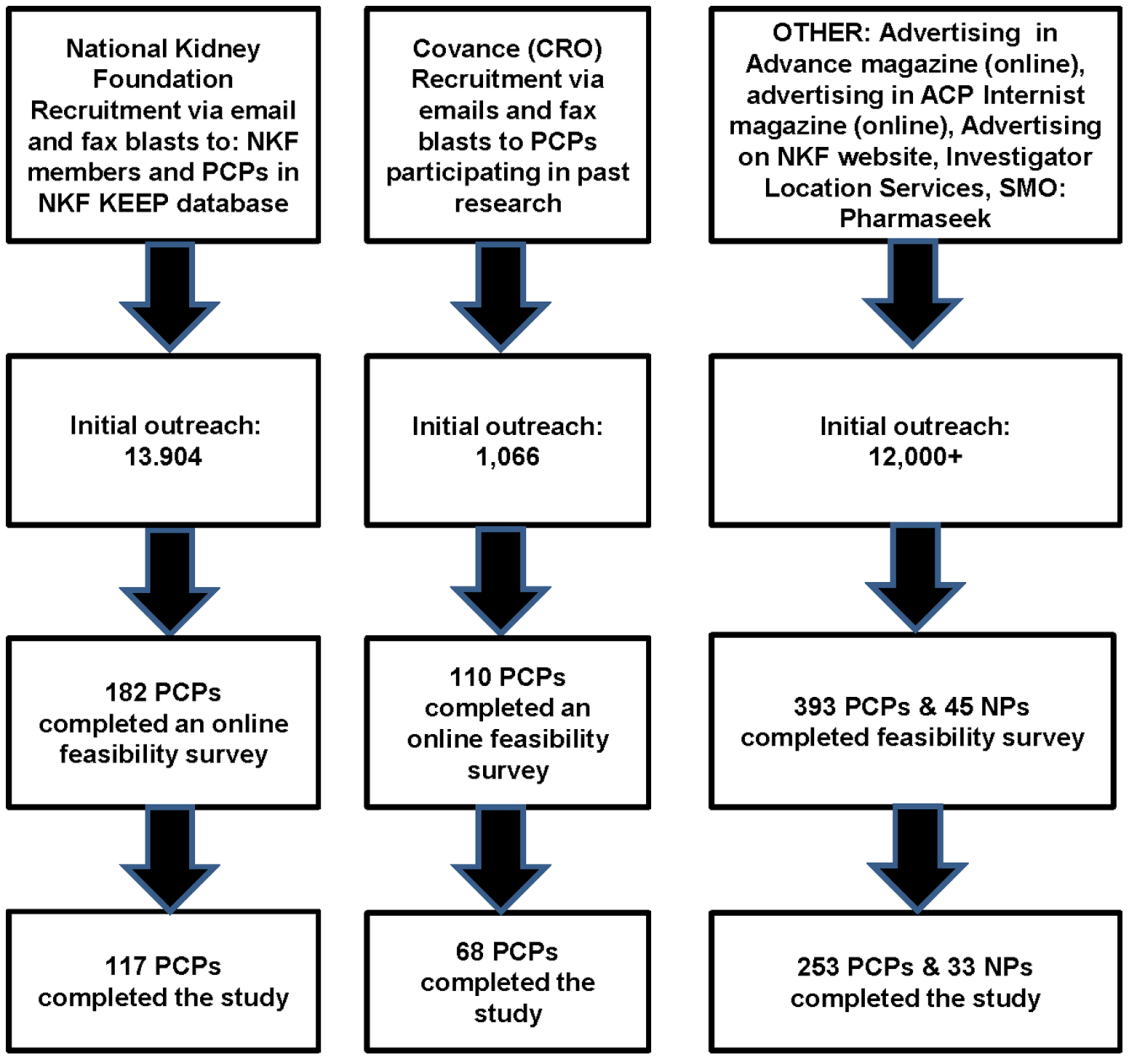
Recruitment Flow Diagram.

This multi-center, observational, cross-sectional study was conducted in 466 primary care practices in a geographic distribution representative of the US between 6/28/11 and 2/06/12. Primary care practitioners were recruited using several mechanisms described in Figure 1, including: advertising in medical journals and primary care physicians in the NKF's Kidney Early Evaluation Program (KEEP) database and the Covance database, the latter of which is comprised of clinicians who previously expressed interest or participated in research with this clinical research organization. On-line magazines were the American College of Physicians (ACP) Internist and Advance for nurse practitioners. Lastly, Pharmaseek, an investigative site network, was also used to identify potential PCP investigators. To ensure that sites reflected the census districts for urban, rural and suburban, providers and a broad range of health insurance plans, a feasibility survey was geographically dispersed to all areas of the country to recruit potential PCPs. A target of 21 individuals with T2DM from each site or PCP was set to achieve a total sample size of approximately 9660 patients. This study was observational in nature without protocol-mandated treatments. Patients enrolled in the study were treated by their PCP according to their usual standard of care. The study was approved by Concordia Clinical Research, an external Institutional Review Board.

Patients 18 years of age or older with T2DM for one year or longer were eligible to participate. Key exclusion criteria included current therapy with peritoneal or hemodialysis or a functioning kidney transplant. A consecutive sampling approach was encouraged to minimize selection biases.

After signing informed consent, each patient underwent a blood draw to determine eGFR, and HbA1c, a urine analysis to detect proteinuria, a urine measurement for ACR, and two patient HRQoL questionnaires. A national laboratory, Quest Diagnostics, was utilized for all study related laboratory measurements. At the time of the Study Visit, a physical examination including measurement of weight and waist circumference was conducted. Data were recorded using a secure online electronic data capture (EDC) system.

### Data analysis

Sample size calculations suggested that approximately 9660 patients with T2DM would need to be enrolled to achieve adequate precision in estimating the rate of undiagnosed CKD by stage.

Estimated glomerular filtration rate was calculated using the study measured value of isotope dilution mass spectrometry-traceable serum creatinine using the CKD-EPI equation [12]. The urine ACR was calculated by dividing urine albumin in mg by urine creatinine in g to approximate the 24 hour urine excretion of albumin [13]. Proteinuria was assessed using the urine analysis dipstick. Qualitative values of trace and above were considered positive. Only a value of “negative” was analysed as negative. The NKF KDOQI guidelines were used to define CKD stage based on the laboratory measures obtained at the time of their study visit.

After the study visit, all patients’ medical records over the prior 15 months were reviewed to determine if their PCP had documented the presence of any stage of CKD in a clinical note or a billing diagnosis. In addition, a review of selected laboratory data and medications prescribed was also completed. Using the study laboratory derived stage of CKD as the “gold standard”, each patient's correct or incorrect designation as having CKD based on clinical notes and billing diagnoses was determined. Based on this evaluation, each patient was described as either a true positive, true negative, false positive or false negative patient (as defined in Table 1).

**Table 1:** Prevalence of Chronic Kidney Disease by Stage based on Estimated Glomerular Filtration Rate and Relevant Laboratory Tests at Study Visit (Patients Who Could be Assessed for Chronic Kidney Disease).

Parameter		CKD by Study Laboratory Results at Study Visit		Normal Kidney Function by Study Laboratory Results at Study Visit	
	Total of Assessed Patients	True Positive	False Negative	True Negative	False Positive
	(N = 9307)^a^	(N = 607)	(N = 4429)	(N = 4213)	(N = 58)
**All patients (%)^a^**	N/A	6.5%	47.6%	45.3%	0.6%
**Stage of CKD at study visit, n(%)^b^**					
No CKD (eGFR ≥60 with no protein excretion)	4271 (45.9)	0	0	4213 (100.0)	58 (100.0)
1 (eGFR ≥90 with abnormal protein excretion)	1038 (11.2)	11 (1.8)	1027 (23.2)	0	0
2 (eGFR: 60–89 with abnormal protein excretion): Mild	1602 (17.2)	79 (13.0)	1523 (34.4)	0	0
3 (eGFR: 30–59): Moderate	2156 (23.2)	389 (64.1)	1767 (39.9)	0	0
4 (eGFR: 15–29): Severe	223 (2.4)	118 (19.4)	105 (2.4)	0	0
5 (eGFR <15 or dialysis): Kidney Failure	17 (0.2)	10 (1.6)	7 (0.2)	0	0
Overall (Stage 2–5): Mild to Kidney Failure	3998 (43.0)	596 (98.2)	3402 (76.8)	0	0
Overall (Stage 1–5)	5036 (54.1)	607 (100.0)	4429 (100.0)	0	0

Abbreviations: ACR  =  albumin/creatinine ratio; CKD  =  chronic kidney disease; eGFR  =  estimated glomerular filtration rate; N  =  number of patients; N/A  =  not applicable.

True Positive: Patients who reported as diagnosed with CKD (on CKD History eCRF page) and with actual presence of CKD based on laboratory results from the Study Visit;

True Negative: Patients who reported as not diagnosed with CKD (on CKD History eCRF page) and without actual presence of CKD based on laboratory results from the Study Visit;

False Positive: Patients who reported as diagnosed with CKD (on CKD History eCRF page) and without actual presence of CKD based on laboratory results from the Study Visit;

False Negative: Patients who reported as not diagnosed with CKD (on CKD History eCRF page) and with actual presence of CKD based on laboratory results from the Study Visit.

a: A total of 9339 patients enrolled in the study, but only 9307 patients had the laboratory test results and medical history records to assess as True Positive, True Negative, False Positive, or False Negative for CKD.

b: If eGFR, urine dipstick, and urine ACR data were all available:

•No CKD: Normal eGFR (≥60 mL/min/1.73 m^2^) with neither positive protein in urine nor urine ACR ≥30 mg/g.

•Stage 1: Normal eGFR (≥90 mL/min/1.73 m^2^) with either positive protein in urine or urine ACR ≥30 mg/g.

•Stage 2: eGFR 60 to 89 mL/min/1.73 m^2^ with either positive protein in urine or urine ACR ≥30 mg/g.

•Stage 3: eGFR  = 30 to 59 mL/min/1.73 m^2^;

•Stage 4: eGFR  = 15to 29 mL/min/1.73 m^2^; and

•Stage 5: eGFR <15 mL/min/1.73 m^2^ (Stages 3, 4, 5 were based on eGFR value alone).

If eGFR, urine dipstick, and urine ACR data were not all available: classify with the worst CKD stage based on the available data from eGFR, urine dipstick, and/or urine ACR.

Among sites that enrolled 10 or more patients, the Q1 (25th percentile), median, and Q3 (75th percentile) sensitivity for diagnosing CKD in patients with CKD established using study related labs were 0, 6.3, and 16.7%. Because this distribution was skewed with the tail to the right, sensitivities were categorized as 0%, >0% to <50%, and ≥50% to compare PCP characteristics.

To assess patients' current state of CKD awareness, patients were asked “Have you ever been told by a doctor or other health care professional that you have a chronic kidney disease (a decreased GFR, or elevated serum creatinine, or weak and/or failing kidneys)?”

Data were summarized overall and among patient subgroups based on true positive, true negative, false positive, and false negative categories. Discrete and ordinal variables were summarized by frequencies and percentages [n (%)], whereas continuous variables were summarized by mean, standard deviation (SD), median, 25th and 75th percentiles, minimum, and maximum.

All analyses were performed using the SAS statistical software package (Version 9.1, Cary, NC).

## Results

### Patient level description

**Figure 2:**
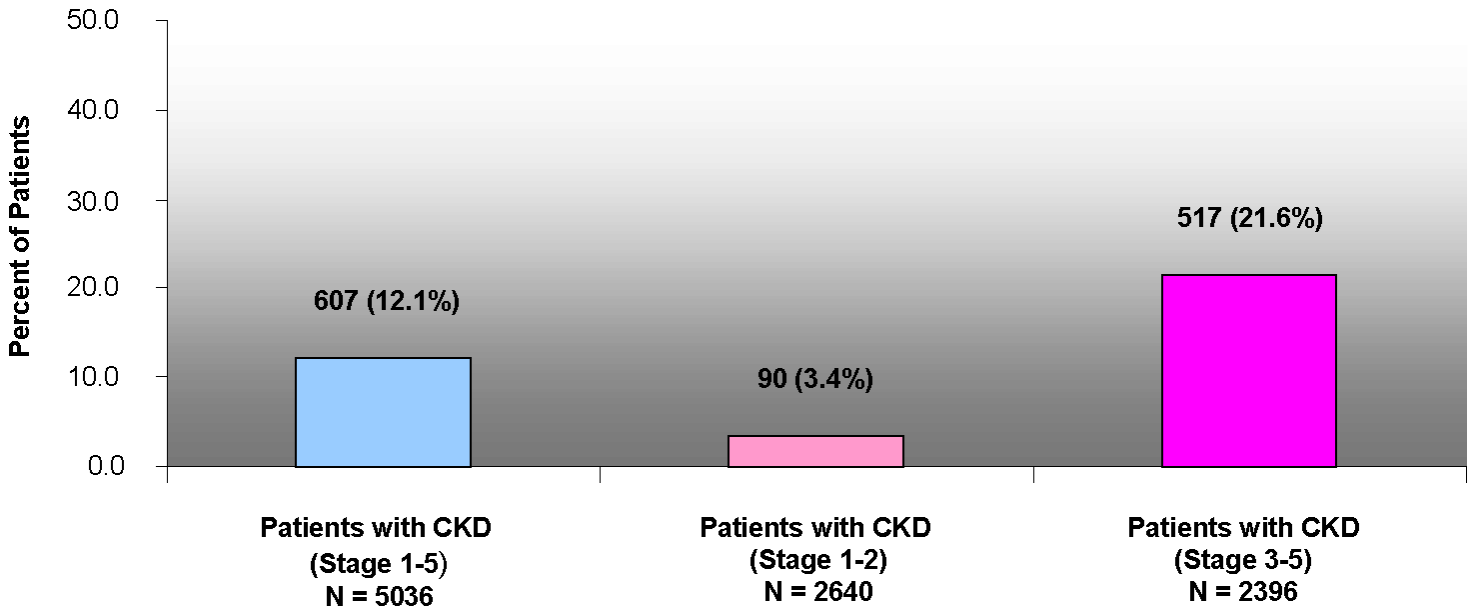
Percent of Patients with CKD.

**Figure 3:**
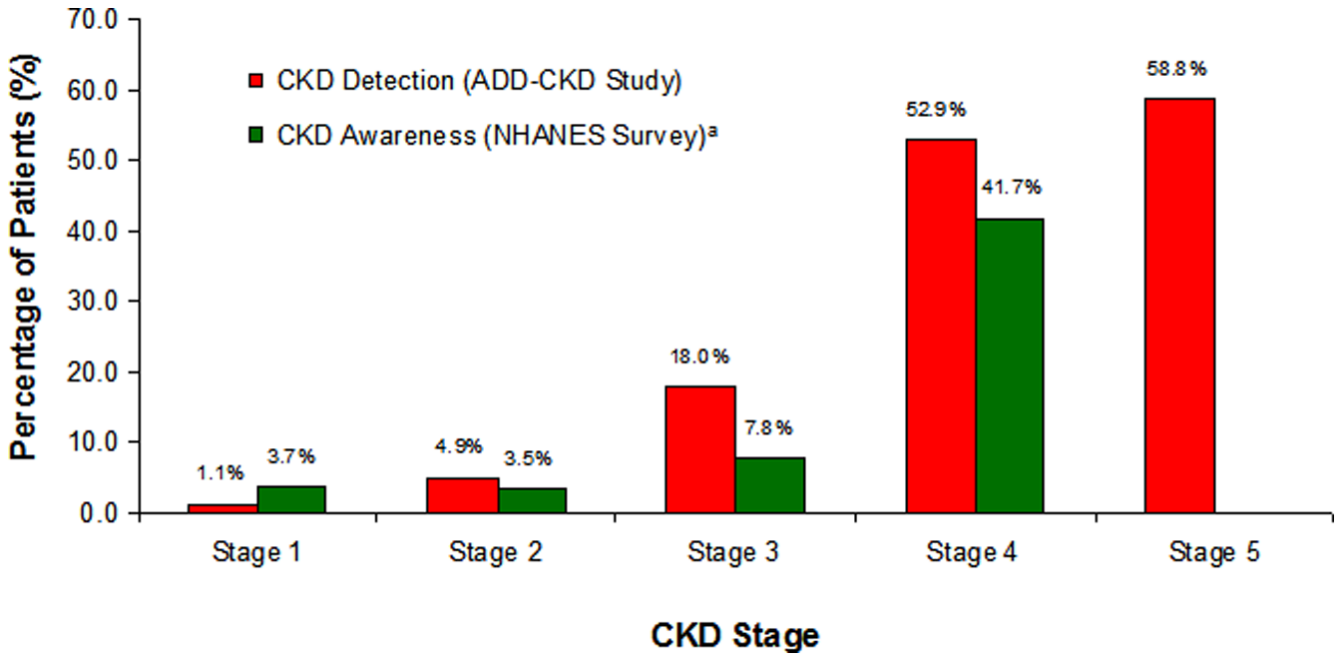
Proportion of Patients with CKD Detected by CKD Stage Prior to Study Visit Compared to Proportion of Patient-reported CKD Awareness.

A total of 9339 patients were enrolled at 466 US practices. Of these, 9307 (99.7%) patients could be assessed for the presence of CKD, and 9204 (98.6%) completed all aspects of the study. Of the 9307 patients with laboratory data, 54.1% had CKD (Table 1). Of the 5036 patients with CKD (patients in the True Positive and False Negative groups), 12.1% of patients with CKD were identified by the PCP prior to study participation (Figure 2). As stage of CKD worsened, the proportion of patients correctly identified as having CKD by the PCP increased (1.1% of Stage 1, 4.9% for Stage 2, 18.0% for Stage 3, 52.9% for Stage 4, and 58.8% for Stage 5 (Figure 3).

When comparing patients with CKD identified prior to study enrollment by their PCP (True Positives) to those with CKD who were not identified as having CKD prior to study enrollment (False Negatives) and to those without CKD (True Negatives and False Positives), there were few differences (Table 2). Gender, race, ethnicity, and body mass index were similar for each of the four groups. The distribution of age shifted toward the older age categories when comparing the True Positive group to the other groups. With respect to smoking history, patients in the True Positive group were less likely to currently smoke and more likely to have previously smoked.

**Table 2:** Summary of Patient Demographics, Other Baseline Characteristics, and Medical History (All Enrolled Patients).

Parameter		CKD by Study Laboratory Results at Study Visit		Normal Kidney Function by Study Laboratory Results at Study Visit	
		True	False	True	False
	Total	Positive	Negative	Negative	Positive
	(N = 9339)^a^	(N = 607)	(N = 4429)	(N = 4213)	(N = 58)
	n (%)	n (%)	n (%)	n (%)	n (%)
**DEMOGRAPHIC AND OTHER BASELINE CHARACTERISTICS**					
**Age categories (years), n (%)^b^**					
18–24	12 (0.1)	0	5 (0.1)	7 (0.2)	0
25–34	114 (1.2)	0	44 (1.0)	68 (1.6)	0
35–44	573 (6.1)	9 (1.5)	227 (5.1)	333 (7.9)	2 (3.4)
45–54	1621(17.4)	31 (5.1)	636 (14.4)	942 (22.4)	9 (15.5)
55–64	2867 (30.7)	121 (19.9)	1256 (28.4)	1462 (34.7)	19 (32.8)
65–74	2717 (29.1)	250 (41.2)	1363 (30.8)	1077 (25.6)	15 (25.9)
≥75	1434 (15.4)	196 (32.3)	897 (20.3)	324 (7.7)	13 (22.4)
Missing	1 (0.0)	0	1 (0.0)	0	0
**Gender, n (%)**					
Male	4580 (49.0)	313 (51.6)	2207 (49.8)	2017 (47.9)	27 (46.6)
Female	4759 (51.0)	294 (48.4)	2222 (50.2)	2196 (52.1)	31 (53.4)
**Race, n (%)**					
White	6972 (74.7)	471 (77.6)	3343 (75.5)	3090 (73.3)	45 (77.6)
Black or African American	1579 (16.9)	93 (15.3)	734 (16.6)	740 (17.6)	10 (17.2)
American Indian or Alaska Native	32 (0.3)	1 (0.2)	13 (0.3)	18 (0.4)	0
Asian, Native Hawaiian, or other Pacific Islander	371 (4.0)	19 (3.1)	166 (3.7)	180 (4.3)	2 (3.4)
Other	379 (4.1)	23 (3.8)	170 (3.8)	184 (4.4)	1 (1.7)
Missing	6 (0.1)	0	3 (0.1)	1 (0.0)	0
**Ethnicity, n (%)**					
Hispanic or Latino	1231 (13.2)	67 (11.0)	560 (12.6)	589 (14.0)	8 (13.8)
Not Hispanic or Latino	8102 (86.8)	540 (89.0)	3866 (87.3)	3623 (86.0)	50 (86.2)
Missing	6 (0.1)	0	3 (0.1)	1 (0.0)	0
**BMI (kg/m^2^)^c^**					
n	9332	606	4427	4211	58
Mean	33.7	33.5	33.8	33.8	33.1
SD	7.6	7.6	7.7	7.5	7.4
**Current smoking habits, n (%)**					
Still smoke	1218 (13.0)	53 (8.7)	572 (12.9)	583 (13.8)	7 (12.1)
Used to smoke	3423 (36.7)	260 (42.8)	1631 (36.8)	1497 (35.5)	21 (36.2)
Missing	4698 (50.3)	294 (48.4)	2226 (50.3)	2133 (50.6)	30 (51.7)
**MEDICAL HISTORY**					
**Patients with self-reported kidney disease, n (%)**					
Yes	676 (7.2)	492 (81.1)	117 (2.6)	31 (0.7)	34 (58.6)
No	8656 (92.7)	115 (18.9)	4308 (97.3)	4181 (99.2)	24 (41.4)
Missing	7 (0.1)	0	4 (0.1)	1 (0.0)	0
**Patients with self-reported hypertension, n (%)**					
Yes	7605 (81.4)	571 (94.1)	3750 (84.7)	3215 (76.3)	48 (82.8)
No	1727 (18.5)	36 (5.9)	675 (15.2)	997 (23.7)	10 (17.2)
Missing	7 (0.1)	0	4 (0.1)	1 (0.0)	0
**Patients with hypercholesterolemia, n (%)**					
Yes	7297 (78.1)	532 (87.6)	3487 (78.7)	3210 (76.2)	45 (77.6)
No	2035 (21.8)	75 (12.4)	938 (21.2)	1002 (23.8)	13 (22.4)
Missing	7 (0.1)	0	4 (0.1)	1 (0.0)	0
**Patients with cardiovascular disease^d^, n (%)**					
Yes	3740 (40.0)	358 (59.0)	1940 (43.8)	1398 (33.2)	30 (51.7)
No	5582 (59.8)	249 (41.0)	2482 (56.0)	2807 (66.6)	28 (48.3)
Missing	17 (0.2)	0	7 (0.2)	8 (0.2)	0

Note: Denominators for percentages are based on the number of enrolled patients.

Abbreviations: BMI  =  body mass index; CKD  =  chronic kidney disease; eCRF  =  estimated glomerular filtration rate; N  =  number of patients; SD  =  standard deviation.

For definitions of True Positive, True Negative, False Positive, and False Negative CKD assessment categories, ***see*** ***Table 1*** ***footnotes.***

a: A total of 9339 patients enrolled in the study, but only 9307 patients had the laboratory test results and medical history records to assess as True Positive, True Negative, False Positive, or False Negative for CKD.

b: Age is calculated as (informed consent date - date of birth)/365.25 and reported as whole years.

c: BMI (kg/m^2^) is calculated as (Weight(kg)/Height(m)^2^)

d: If patient checked "Yes" for heart angina, heart attack, heart bypass surgery, heart angioplasty, stroke, heart failure, abnormal heart rhythm, or coronary artery disease on Medical History eCRF page.

With respect to the self-reported presence of CKD, few patients in the True Negative and False Negative Groups reported the presence of kidney disease (0.7 and 2.6%, respectively) while the majority of patients in the True Positive group reported knowing that they had kidney disease (81.1%). The proportion of patients reporting concurrent comorbid conditions such as hypertension, hypercholesterolemia, and cardiovascular disease was consistently greater in the True Positive group.

### Primary care practitioner assessment of CKD

Of the 445 PCPs who enrolled at least 10 patients, 19 (4.3%) had ≥50% likelihood of identifying patients with CKD, 217 (48.8%) had a likelihood of <50%, and 209 (47.0%) didn't identify any of their CKD patients (had a 0% sensitivity).

The patient specific demographic and clinical factors that influenced clinicians to screen for CKD were similar among the groups based on sensitivity (Table 3). PCPs in the group with ≥50% sensitivity were more likely to consider the presence of cardiovascular disease (78.9% as compared to 71.0% and 69.9% in the groups with lower sensitivity) and a family history of kidney disease (94.7% as compared to 78.3% and 78.0% in the groups with lower sensitivity) as factors that would influence their desire to screen. PCPs in all three categories of sensitivity reported similar frequencies of the use of eGFR, urine analysis, and urine ACR tests to screen for kidney disease with modes at 3, 6, and 12 months.

**Table 3:** Primary Care Provider Self-Reported Clinical Practice Patterns of Screening Frequency and Factors that Influence Their Likelihood to Screen Patients for Chronic Kidney Disease (Selected Parameters of Interest).

Parameter	Sensitivity^a^		
	0%	>0 to <50%	≥50%
	(N = 209)	(N = 217)	(N = 19)
**Demographic and clinical factors that influence screening for CKD** b **, n (%)**			
Age	161 (77.0)	160 (73.7)	16 (84.2)
Gender	65 (31.1)	54 (24.9)	6 (31.6)
Race	115 (55.0)	107 (49.3)	9 (47.4)
Presence of T2DM	204 (97.6)	213 (98.2)	19 (100.0)
Presence of CVD	146 (69.9)	154 (71.0)	15 (78.9)
Presence of Hypertension	189 (90.4)	201 (92.6)	18 (94.7)
Family History of Kidney Disease	163 (78.0)	170 (78.3)	18 (94.7)
Other	16 (7.7)	15 (6.9)	3 (15.8)
**Frequency of eGFR test for screening for CKD, n (%)**			
Every 1 Month	2 (1.0)	3 (1.4)	1 (5.3)
Every 2 Months	1 (0.5)	1 (0.5)	0 (0.0)
Every 3 Months	56 (26.8)	64 (29.5)	5 (26.3)
Every 4 Months	15 (7.2)	15 (6.9)	1 (5.3)
Every 5 Months	0 (0.0)	1 (0.5)	0 (0.0)
Every 6 Months	69 (33.0)	73 (33.6)	10 (52.6)
Every 9 Months	1 (0.5)	0 (0.0)	0 (0.0)
Every 12 Months	29 (13.9)	28 (12.9)	1 (5.3)
Missing	36 (17.2)	32 (14.7)	1 (5.3)
**Frequency of urinalysis test for screening for CKD, n (%)**			
Every 1 Month	5 (2.4)	5 (2.3)	1 (5.3)
Every 2 Months	0 (0.0)	2 (0.9)	1 (5.3)
Every 3 Months	30 (14.4)	34 (15.7)	2 (10.5)
Every 4 Months	6 (2.9)	8 (3.7)	0 (0.0)
Every 6 Months	36 (17.2)	30 (13.8)	0 (0.0)
Every 12 Months	61 (29.2)	50 (23.0)	3 (15.8)
Missing	71 (34.0)	88 (40.6)	12 (63.2)
**Frequency of urine ACR test for screening for CKD, n (%)**			
Every 1 Month	1 (0.5)	2 (0.9)	0 (0.0)
Every 2 Months	0 (0.0)	2 (0.9)	0 (0.0)
Every 3 Months	17 (8.1)	22 (10.1)	2 (10.5)
Every 4 Months	4 (1.9)	7 (3.2)	0 (0.0)
Every 5 Months	2 (1.0)	0 (0.0)	0 (0.0)
Every 6 Months	37 (17.7)	29 (13.4)	5 (26.3)
Every 9 Months	1 (0.5)	0 (0.0)	0 (0.0)
Every 12 Months	66 (31.6)	75 (34.6)	8 (42.1)
Every 24 Months	1 (0.5)	0 (0.0)	0 (0.0)
Missing	80 (38.3)	80 (36.9)	4 (21.1)
**eGFR (mL/min/1.73 m^2^) value that would indicate a patient has CKD, n (%)**			
<30	7 (3.3)	12 (5.5)	0 (0.0)
<40	10 (4.8)	0 (0.0)	0 (0.0)
<45	4 (1.9)	3 (1.4)	0 (0.0)
<50	24 (11.5)	21 (9.7)	3 (15.8)
<55	4 (1.9)	9 (4.1)	0 (0.0)
<59	7 (3.3)	13 (6.0)	1 (5.3)
<60	120 (57.4)	130 (59.9)	14 (73.7)
<65	1 (0.5)	2 (0.9)	0 (0.0)
<70	1 (0.5)	2 (0.9)	0 (0.0)
<75	2 (1.0)	1 (0.5)	0 (0.0)
<80	2 (1.0)	5 (2.3)	0 (0.0)
<90	19 (9.1)	15 (6.9)	1 (5.3)
<100	4 (1.9)	2 (0.9)	0 (0.0)
Missing	4 (1.9)	2 (0.9)	0 (0.0)
**Proteinuria in urinalysis value that would indicate a patient has CKD, n (%)**			
+1	110 (52.6)	93 (42.9)	12 (63.2)
+2	44 (21.1)	56 (25.8)	5 (26.3)
+3	24 (11.5)	23 (10.6)	2 (10.5)
+4	5 (2.4)	6 (2.8)	0 (0.0)
trace	22 (10.5)	36 (16.6)	0 (0.0)
Missing	4 (1.9)	3 (1.4)	0 (0.0)
**ACR in urine (mg/gram) value that would indicate a patient has CKD, n (%)**			
>0	10 (4.8)	8 (3.7)	0 (0.0)
>1	5 (2.4)	7 (3.2)	1 (5.3)
>2	8 (3.8)	7 (3.2)	0 (0.0)
>3	3 (1.4)	4 (1.8)	0 (0.0)
>4	0 (0.0)	1 (0.5)	0 (0.0)
>5	6 (2.9)	4 (1.8)	0 (0.0)
>10	4 (1.9)	2 (0.9)	0 (0.0)
>15	1 (0.5)	2 (0.9)	0 (0.0)
>16	0 (0.0)	1 (0.5)	0 (0.0)
>20	7 (3.3)	6 (2.8)	1 (5.3)
>23	2 (1.0)	0 (0.0)	1 (5.3)
>25	0 (0.0)	4 (1.8)	0 (0.0)
>29	2 (1.0)	0 (0.0)	0 (0.0)
>30	114 (54.5)	115 (53.0)	11 (57.9)
>31	0 (0.0)	1 (0.5)	0 (0.0)
>35	3 (1.4)	2 (0.9)	1 (5.3)
>40	1 (0.5)	1 (0.5)	0 (0.0)
>50	5 (2.4)	4 (1.8)	0 (0.0)
>60	2 (1.0)	1 (0.5)	0 (0.0)
>80	0 (0.0)	1 (0.5)	0 (0.0)
>84	1 (0.5)	0 (0.0)	0 (0.0)
>100	4 (1.9)	5 (2.3)	0 (0.0)
>150	2 (1.0)	1 (0.5)	0 (0.0)
>200	5 (2.4)	2 (0.9)	0 (0.0)
>250	1 (0.5)	1 (0.5)	0 (0.0)
>299	1 (0.5)	1 (0.5)	0 (0.0)
>300	14 (6.7)	30 (13.8)	3 (15.8)
Missing	8 (3.8)	6 (2.8)	1 (5.3)
**Protein/creatinine ratio on urine (mg/gram) value that would indicate a patient has CKD, n (%)**			
>0	15 (7.2)	11 (5.1)	0 (0.0)
>1	6 (2.9)	8 (3.7)	1 (5.3)
>2	14 (6.7)	18 (8.3)	2 (10.5)
>3	9 (4.3)	12 (5.5)	1 (5.3)
>4	0 (0.0)	3 (1.4)	0 (0.0)
>5	8 (3.8)	2 (0.9)	0 (0.0)
>6	0 (0.0)	0 (0.0)	1 (5.3)
>9	0 (0.0)	0 (0.0)	1 (5.3)
>10	8 (3.8)	7 (3.2)	0 (0.0)
>15	2 (1.0)	5 (2.3)	0 (0.0)
>16	1 (0.5)	0 (0.0)	0 (0.0)
>20	5 (2.4)	7 (3.2)	0 (0.0)
>24	0 (0.0)	0 (0.0)	1 (5.3)
>25	0 (0.0)	1 (0.5)	0 (0.0)
>30	49 (23.4)	52 (24.0)	3 (15.8)
>35	4 (1.9)	2 (0.9)	1 (5.3)
>44	1 (0.5)	0 (0.0)	0 (0.0)
>45	3 (1.4)	2 (0.9)	1 (5.3)
>50	6 (2.9)	5 (2.3)	0 (0.0)
>60	0 (0.0)	2 (0.9)	0 (0.0)
>80	0 (0.0)	1 (0.5)	0 (0.0)
>84	4 (1.9)	5 (2.3)	0 (0.0)
>85	2 (1.0)	0 (0.0)	0 (0.0)
>90	1 (0.5)	0 (0.0)	0 (0.0)
>100	9 (4.3)	6 (2.8)	1 (5.3)
>120	0 (0.0)	0 (0.0)	1 (5.3)
>130	0 (0.0)	1 (0.5)	0 (0.0)
>150	3 (1.4)	6 (2.8)	0 (0.0)
>200	25 (12.0)	22 (10.1)	2 (10.5)
>250	0 (0.0)	1 (0.5)	0 (0.0)
>275	0 (0.0)	1 (0.5)	0 (0.0)
>299	1 (0.5)	0 (0.0)	0 (0.0)
>300	21 (10.0)	28 (12.9)	1 (5.3)
Missing	12 (5.7)	8 (3.7)	2 (10.5)

Abbreviations: ACR  =  albumin/creatinine ratio; CKD  =  chronic kidney disease; CVD  =  cardiovascular disease; eGFR  =  estimated glomerular filtration rate; N  =  number of patients; T2DM  =  type 2 diabetes mellitus.

a . Only sites with at least 10 enrolled patients were included for this analysis. Sensitivity for each investigator site was defined as (No. of True Positive)/(No. of True Positive + No. of False Negative) x 100%.

b . Provider could check more than one category.

When asked what eGFR value indicates that a patient has CKD, the greatest proportion of PCPs reported an eGFR of <60 mL/min/1.73 m^2^ was the threshold value with a smaller number reporting a threshold eGFR value of <90 mL/min/1.73 m^2^. While there were no significant differences among groups overall, a small but significant proportion of clinicians in each category reported <50 mL/min/1.73 m^2^ as the threshold. There were too few nurse practitioners, 33, to allow for meaningful comparisons versus physicians.

When asked what level of urine albumin excretion indicates that a patient has CKD, the greatest proportion of PCPs (53.9%) reported an ACR of>30 mg/gm as the threshold value above which a patient has CKD. The proportion reporting this value was roughly equivalent among sensitivity categories, while the remainder of PCPs reported values that were higher or lower than the expected value. When asked what level of proteinuria on dipstick urine analysis indicates that a patient has CKD, more than half of all providers reported trace or +1. However, significant proportions that did not differ among categories of sensitivity also reported thresholds of +2 or +3.

During the 15 months prior to study participation, 15.2% of the patients enrolled in this study did not have an eGFR test performed and 31.0% did not have a test for either proteinuria or ACR (51.4% did not have a test for proteinuria and 52.9% did not have an urine ACR measured).

## Discussion

This study quantified the degree to which CKD was recognized in a population of patients at a higher risk due to the presence of T2DM. More than half of participants had CKD as manifested by changes in urine protein excretion, a decreased eGFR, or both. However, among those patients with CKD only 12.1% had their CKD documented as either a diagnosis code or description in the fifteen month chart review.

Clinicians were more successful in recognizing CKD in more advanced stages (e.g., 3 and 4), but still missed nearly half of patients with stage 4 CKD. Fewer than 5% of clinicians achieved a sensitivity of ≥50% with nearly half of all PCPs not applying the diagnosis in any of their patients with CKD. Few differences were identified in approaches to screening for CKD, and interpretations of the screening test results closely resembled the current clinical practice guidelines. PCPs were consistent with the guidelines with regard to the measurement of serum creatinine (eGFR). However, in spite of their acknowledgment of the importance of assessing urine for abnormal levels of protein excretion, 69% of patients had proteinuria tests in the 15 months prior to participation.

The prevalence of CKD found in this cohort is similar to that described among all patients with T2DM in the US (NHANES dataset) [3]. These findings complement the work of Plantinga et al [14]. in assessing patient awareness of CKD at each stage of the disease. Using the NHANES Survey 1999–2004, the proportion of patients who answered yes to the question "have you ever been told that you have weak or failing kidneys" were quantified by stage of kidney disease. Among patients with Stages 1–3 CKD, only 3.7, 3.5, and 7.8% of people with each stage were aware of their CKD (Figure 3). While the proportion of people aware of their CKD rose in Stage 4, it remained low at 41.7%. While differences in study design between the analysis conducted by Plantinga et al [14]. and the cohort described here exist, it is also noteworthy that the proportions of patients with CKD identified by their PCP as having CKD in this study were similar to the proportions of patients aware of their CKD in the analysis by Plantinga. The fact that approximately half of patients were either aware of or diagnosed with CKD among patients with Stage 4 disease in both studies is consistent with USRDS data demonstrating that half of patients beginning dialysis do not see a nephrologist prior to initiation [2].

The lowest proportions of patients were identified in stages with relatively preserved eGFR where CKD is defined by the presence of proteinuria/albuminuria, corresponding to lower urinary testing. Multiple studies suggest that proteinuria is a powerful tool to risk stratify patients not only for the progression of their kidney disease but also for cardiovascular and all-cause mortality [15], [16], [17]. Specific to persons with T2DM, interventions focused on lowering proteinuria prolong both renal and patient survival [18], [19], [20]. Observational data suggests that proteinuria is one of the most powerful markers of overall survival for population based groups at risk than other well-known laboratory tests such as LDL cholesterol [17]. The results presented indicate that while clinicians have an understanding of when and how to screen for CKD, the actual tests required to diagnose CKD with a particular focus on the assessment of abnormal protein excretion, are not being performed at the frequency that they perceive to be their goal. Given the emphasis on proteinuria testing in both the ADA [5] as well as the NKF KDOQI guidelines [6], these results suggest that this noninvasive assessment is currently underutilized. Patient-related factors could also have contributed to this finding, such as missed appointments or not providing an ordered urine test.

This study provides insight into the key points in the delivery of healthcare where identification of CKD can be improved; however, it is not without limitation that may affect the accuracy of the estimates. All stages CKD could have been overestimated, since the single study assessment of ACR and eGFR was not confirmed for a period of>3 months as recommended by CKD clinical practice guidelines. Moreover, the ADA recommends 3 determinations of albuminuria over a six month interval. Thus, the clinician under-detection of CKD is not surprising based on the test results available for review. However, there was indeed remarkable underutilization of the eGFR in 15.2% and ACR in 52.9% tests in the 15 month retrospective data review, despite annual recommendations for people with type-2 diabetes from the NKF [4] and ADA [5]. While investigators were instructed to enroll consecutive patients, this design was one of a convenience sample of patients. Investigators were aware that the objective of the study was to clarify the rate at which they appropriately identified patients with CKD. An important discussion point is the consideration of whether or not the presence of a billing code or a documented diagnosis of CKD in the medical record truly represents valid surrogates for provider knowledge of CKD in an individual patient. This documentation was used as the metric in this study because of its objective nature and feasibility. Supporting the assumption that documentation of CKD signifies recognition of its presence is the marked difference in patient knowledge of their own CKD between True Positives and False Negatives. This difference in CKD awareness (81.1 vs. 2.6%, respectively) supports that documentation and communication of the problem to the patient are associated and that failure to document likely reflects a true lack of recognition. This is the first study to demonstrate detection of CKD by the clinician is associated with patient awareness. Although this finding is intuitively obvious, this suggests interventions to enhance PCP detection will also enhance patient awareness. This study demonstrates an under recognition of the presence of CKD among persons with the T2DM that may be mediated more through a lack of application of best practices, as defined by clinical practice guidelines, rather than lack of knowledge of these guidelines. Given the mortality risk associated with CKD and therapies that can reduce this risk, efforts focused on increasing identification are essential. Future design of questionnaires used in NHANES or other studies should consider questions that reflect awareness of albuminuria/proteinuria in addition to “weak or failing kidneys”. These data suggest that efforts must be instituted not only in the general public at risk with T2DM but also among PCPs, with future research focused on a greater understanding of the perceived obstacles to screening. Finally, educational initiatives focusing on the utility of testing for CKD, with an emphasis on proteinuria, should be tested to identify strategies that result in greater awareness that translates to earlier interventions to improve both renal and cardiovascular risk.
